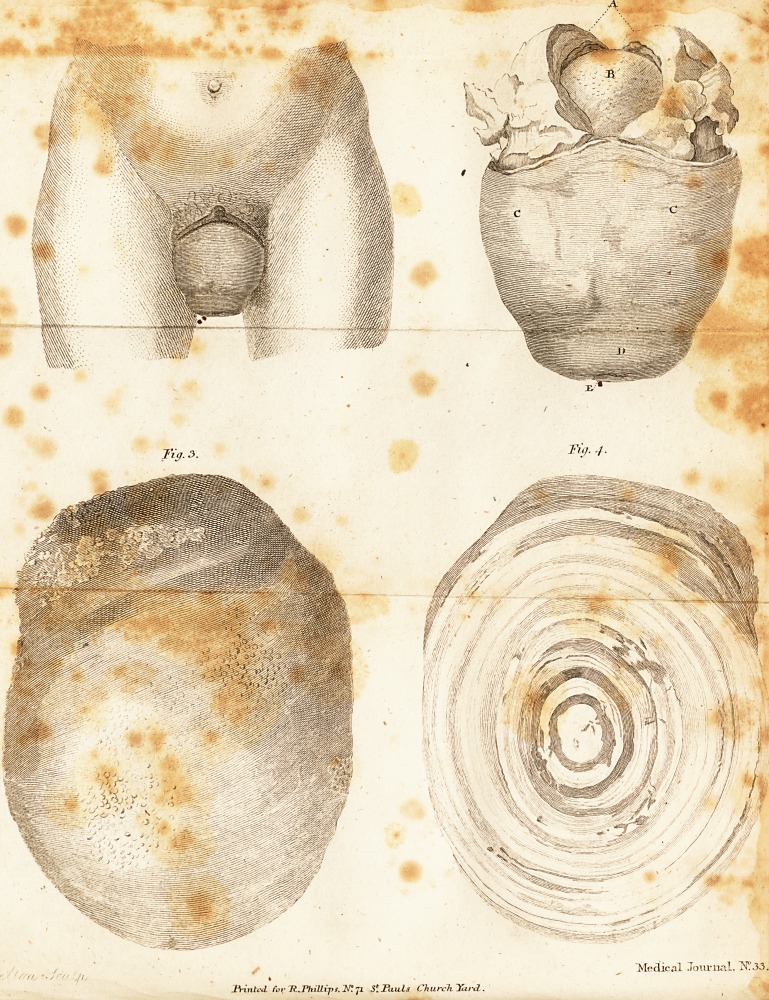# Mr. Paget, on an Extraordinary Calculus

**Published:** 1801-11

**Authors:** Tho. Paget

**Affiliations:** Leicester


### *To the Editors of the Medical and Physical Journal.*

GENTLEMEN,

THE following case, which lately occurred at the Infirmary in this place, being, as I conceive, highly interesting to the pathologist, as well as useful to the practitioner in surgery, I make no apology for communicating it to the public through the medium of your Journal. I am, &c.

*Leicester, Sept.* 16, 1801. THO. PAGET.

---

MARGARET PEAT, aged 47, was admitted into the Infirmary on account of a large substance protruding from the pudenda, and which was supposed to be a prolapsus uteri. She had been married several years, but had never been pregnant

The disease had continued nearly in the same state, but increasing in magnitude for eight or nine years, previous to which she had, for a considerable time, experienced a sensation of *bearing down*, but nothing had appeared externally. At the time she was admitted, the tumour was as large as a new-born infant's head, very sore to the touch, and inflamed on the surface, especially on that part of it which was situated under the meatus urinarius, where it was much excoriated by the urine; it felt very heavy, and communicated a sensation on being handled, as if there was a small quantity of a fluid between the external covering and a harder substance, which appeared to form the bulk of the tumour within. The upper part of it extended from the meatus urinarius quite to the extremity of the rectum, which was pressed forcibly by it against the os coccygis; towards the lower part it became gradually of smaller diameter, and near the extremity was a substance apparently distinct from the main body, and which was nearly insensible, terminating in an opening at the apex, which was evidently the os tincæ, and from which she had regularly menstruated until the last eight or nine months of her existence. The longest diameter of the tumour was about eight inches, and it was incapable of being returned in any degree within the pelvis. It is remarkable, that notwithstanding this formidable disease had continued such a length of time, she had never consulted any medical practitioner on her case, and, until a few months previous to her admission, had performed her household business as usual, and even went out to harvest work the summer before. At the time she came to the Infirmary she suffered extreme pain, which came on at intervals similar to the pains of labour; and was generally attended with a troublesome prolapsus ani, by which her distress was greatly augmented. Her urine, which was highly purulent, was only evacuated during the pains, and that in small quantity. On attempting to introduce a catheter, with a view of relieving the suppression of urine, and of ascertaining whether the bladder had any share in the disease, I found an obstruction to its entrance; the meatus, though wide at the external orifice, took a sudden turn, which rendered all efforts to pass even the smallest catheters or bougies ineffectual, and the pain was greatly aggravated by every attempt of that kind. Various medicines and applications of the palliative kind were made use of with little effect, and, after four or five weeks, death put an end to her sufferings.

On examination of the parts after death, the phenomena attending the disease became very easy of solution. The inferior extremity of the tumour terminating in the os tincæ, evidently shewed that the uterus formed at least a part of its bulk; and, accordingly, accordingly, that viſcus was found of its natural ſize, and perfectly free from diſeaſe, forming the lower inſenſible part of the protruded body. The upper portion was compoſed of the urinary bladder reflected backwards, and the whole was covered with the vagina inverted. The bladder was much diſeaſed, and in ſome parts increaſed to nearly an inch in thickneſs, the inner ſurface being covered with purulent matter, which was alſo contained in great abundance within the rugæ. The bladder was found to contain one enormous calculus, beſides innumerable ſmaller ones, ſome as large as peas, and others ſmaller. The large ſtone was of a light aſh colour, rough on its ſurface, of a flattened oval ſhape, and when firſt taken out weighed *twenty-seven ounces.** On its external ſurface was a ſulcus, occaſioned by the preſſure of the diſtended labia pudendi; and the internal diſtribution of the laminæ ſhews that the upper part of the ſtone was formed after the bladder was rendered incapable of farther lateral diſtenſion.

---

EXPLANATION of the FIGURES.

Fig. 1. The appearance of the protruded part in the living ſubject.

2. The parts as they appeared on diſſection.

A. The diſeaſed and thickened ſubſtance of the bladder.

B. The calculus in ſitu.

C C. The inverted vagina.

D. The body of the uterus inveſted with the inverted vagina.

E. The os tincæ.

3. The external appearance of the calculus, of its natural ſize.

4. The ſtone, ſawn through longitudinally, to ſhew the diſtribution of the laminæ.

---

### *The Case of a Lady at New York, North America, as mentioned in Letter to a Friend, dated May* 23, 1801. *Communicated by* D. Cox, *Eſq.*

I HAVE been greatly afflicted all laſt Winter with a complaint in my eyes of a ſingular nature; an inſtance of which none of the Phyſicians have ever heard or read of. After ſleeping an hour or two, on opening my eyes, which I cannot reſiſt,

---

* This ſtone, together with the diſeaſed bladder, uterus, &c. I have preſented to Mr. Aſtley Cooper, lecturer in anatomy and ſurgery, at St. Thomas's Hoſpital.

Fig. 3.

Fig. 4.

Skelton Sculp.

Printed for R. Phillips, Nº 71 St. Pauls Church Yard.

## Figures and Tables

**Fig. 3. Fig. 4. f1:**